# Radiation dose affected by mammographic composition and breast size: first application of a radiation dose management system for full-field digital mammography in Korean women

**DOI:** 10.1186/s12957-017-1107-6

**Published:** 2017-02-02

**Authors:** Ji Eun Baek, Bong Joo Kang, Sung Hun Kim, Hyun Sil Lee

**Affiliations:** Department of Radiology, Seoul St. Mary’s Hospital, College of Medicine, The Catholic University of Korea, 222, Banpo-daero, Seocho-gu, Seoul, 06591 Republic of Korea

**Keywords:** Radiation dose, Digital mammography, FFDM

## Abstract

**Background:**

Relative to Western women, Korean women show several differences in breast-related characteristics, including higher rates of dense breasts and small breasts. We investigated how mammographic composition and breast size affect the glandular dose during full-field digital mammography (FFDM) in Korean women using a radiation dose management system.

**Methods:**

From June 1 to June 30, 2015, 2120 FFDM images from 560 patients were acquired and mammographic breast composition and breast size were assessed. We analyzed the correlations of patient age, peak kilovoltage (kVp), current (mAs), compressed breast thickness, compression force, mammographic breast composition, and mammographic breast size with the mean glandular dose (MGD) of the breast using a radiation dose management system. The causes of increased radiation were investigated, among patients with radiation doses above the diagnostic reference level (4th quartile, ≥75%).

**Results:**

The MGD per view of 2120 images was 1.81 ± 0.70 mGy. In multivariate linear regression analysis, age was negatively associated with MGD (*p* < 0.05). The mAs, kVp, compressed breast thickness, and mammographic breast size were positively associated with MGD (*p* < 0.05). The “dense” group had a significantly higher MGD than the “non-dense” group (*p* < 0.05). Patients with radiation dose values above the diagnostic reference value had large breasts of dense composition.

**Conclusions:**

Among Korean women, patients with large and dense breasts should be more carefully managed to ensure that a constant radiation dose is maintained.

## Background

The use of imaging modalities that utilize ionizing radiation has become increasingly common. Consequently, there is growing concern about the risk of radiation exposure [1, 2]. The cancer risk associated with radiation exposure is generally extrapolated from the long-term follow-up of Japanese atomic bomb survivors from Hiroshima and Nagasaki [3–6]. Regarding the radiation dose, there is a general consensus on the importance of radiation dose management for patient safety. Thus, clinicians are attempting to reduce the radiation dose for patient safety. Several software programs are available to aggregate and analyze data regarding radiation dose management. A radiation dose management system could help radiologists optimize protocols, reduce the radiation dose, and perform quality management.

With respect to mammography, it is well known that the risk of radiation-induced breast cancer is not a deterrent to the mammographic screening of women over 40 years of age; moreover, several studies of Western women have indicated that when woman-years of life are considered, the benefits of earlier detection are particularly pronounced [7–9]. From another perspective, there is notable concern regarding radiation doses from mammography and the risk of radiation-induced breast cancer resulting from the use of mammography for periodic screening. Women may undergo many mammograms during their lifetimes.

Relative to Western women, Korean women show several differences in breast-related characteristics, including higher rates of dense breasts and small breasts as well as a younger age of breast cancer onset [10–12]. Therefore, this cross-sectional study of Korean women was conducted to evaluate the factors correlated with radiation dose, including mammographic breast composition and mammographic breast size. Moreover, this study describes the first application of a commercial radiation dose management system (Radimetrics™) for full-field digital mammography (FFDM) in Korean women. Using this system, we collected multiple, uncomplicated measurements from patients who underwent mammography and evaluated the factors associated with increased radiation dose during FFDM in Korean women.

The purposes of the present study were to investigate how mammographic composition and breast size affected the glandular dose during full-field digital mammography (FFDM) in Korean women, to evaluate the usefulness of the implemented software (radiation dose management system) for collecting various data from mammography patients, and to determine the factors associated with increased radiation dose during FFDM in Korean women.

## Methods

Our institutional review board approved this prospective study and waived the requirement for informed patient consent. We collected mammographic images acquired in June 2015 from two FFDM units [Mammomat Inspiration (Siemens; Erlangen, Germany) and Selenia (Hologic; Bedford, MA, USA)]. The Mammomat Inspiration unit was used from January 1, 2009, and the Selenia unit was used from August 1, 2006. We excluded spot compression and magnification views, mammography-guided localization images, specimen mammography images, and images of stereotactic biopsies. We also excluded images from male patients. Multiple images obtained over time for individual participants were not included in this study. Bilateral or unilateral mammography with routine craniocaudal (CC) and mediolateral oblique (MLO) views of each participant were included.

From June 1 to June 30, 2015, 2321 FFDM images were acquired and sent to PACS and the server of the radiation dose management system. After the exclusion of 201 images (which were spot compression and magnification views; generated for mammography-guided localization, specimen mammography, or stereotactic biopsy; and/or from male patients), 2120 images from 560 patients who underwent bilateral or unilateral mammography with routine CC and MLO views remained. Only these remaining images were included in our study.

All images were assessed for breast composition by two radiologists, with evaluations determined by consensus (BJE, 4 years of experience; BJK, 15 years of experience). The breast composition reported between two radiologists was approximately agreed upon (Gamma, 0.995; asymptotic standard error, 0.002; approx. *T* ^b^, 62.254). In cases for which different breast compositions were chosen, the result was determined by consensus. Breast composition was visually and subjectively assessed based on the Breast Imaging Reporting and Data System (BI-RADS) criteria, which are as follows: (a) almost entirely fatty, (b) scattered areas of fibroglandular density, (c) heterogeneously dense, and (d) extremely dense. Breasts assessed as A or B were categorized as “non-dense”, and breasts assessed as C or D were categorized as “dense”.

Using the posterior nipple line, the mammographic size of the breast was also assessed by one radiologist (BJE, 4 years of experience). The radiologist measured the distance of a line drawn perpendicular from the nipple to the pectoralis muscle on the MLO view. We calculated the average of the bilateral values for bilateral mammograms and used only the ipsilateral value for unilateral mammograms.

We performed data collection and analysis of the mammographic data using a current radiation dose management system [Radimetrics™ Enterprise Platform (Bayer HealthCare, Whippany, NJ)]. The utilization data, parameters, and dose reports were sent to the conventional picture archiving and communication system (PACS) and the server of the radiation dose management system. The radiation dose management system extracted the mean glandular dose (MGD) value from the Digital Imaging and Communications in Medicine (DICOM) tag “Organ Dose (0040, 0316)”, as defined in the DICOM Standard, and presented it to the application for data aggregation for analysis. The radiation dose management system showed the MGD per laterality (left or right) of the breast according to the “L” or “R” value in the image level DICOM tag “Laterality (0020, 0062)” and summed the MGD values within the same laterality. No other calculation was performed in the radiation dose management system. Data regarding peak kilovoltage (kVp), current (mAs), compressed breast thickness, and breast compression force were also extracted from the DICOM tags of each image and used for analysis. We reviewed the data using our personal computers by accessing the web server of the radiation dose management system.

In addition, we investigated the history of breast-conserving surgery of the patients.

We analyzed the correlations of age at mammography and mammographic parameters, such as kVp, mAs, compressed breast thickness, mammographic breast size, and breast compression force, with the MGD of the breast using univariate and multivariate linear regression analyses. We also determined the relationship between mammographic breast composition and MGD.

We divided the mammographic images into two groups as follows: images with doses above the diagnostic reference level (4th quartile, ≥75%) for the MGD and images with doses below the diagnostic reference level (1st to 3rd quartile, <75%) for the MGD. We used the MGD values of mammograms conducted during June 2015 at our hospital to determine this diagnostic reference level. Additionally, we investigated whether factors such as age, mammographic parameters (kVp, mAs, compressed breast thickness, mammographic breast size and breast compression force), and mammographic breast composition were significantly correlated with doses above the diagnostic reference level (4th quartile, ≥75%). We analyzed the data using univariate and multivariate logistic regression analyses and independent Student’s *t* tests. All statistical analyses were performed using the software package SAS Enterprise Guide 5.1 (SAS Institute, Inc., Cary, NC) or R software version 2.15.3 (R Foundation for Statistical Computing, Vienna, Austria; www.r-project.org).

## Results

From June 1 to June 30, 2015, 2120 consecutive images from 560 patients who underwent bilateral or unilateral mammography with routine CC and MLO views were included. The mean age of the 560 patients at mammography was 52.3 years. The mean mammographic size of the breast per person was 82.01 mm. When the 560 patients were stratified according to mammographic breast composition, 183 patients were included in the “non-dense” group (32.68%), and 377 patients were included in the “dense” group (67.32%). Among the 2120 included images, 260 were obtained from patients with a history of ipsilateral breast-conserving surgery (12.26%). The average MGD per view was 1.81 mGy (Table 1).

**Table 1:** Patient characteristics

Person (*n* = 560)		View (number = 2120)	
Age (years)	52.30 ± 10.72	Breast-conserving surgery	
Mammographic breast size (mm)	82.01 ± 22.22	No	1860 (87.78%)
Mammographic breast composition		Yes	260 (12.26%)
Non-dense	183 (32.68%)	Peak kilovoltage (kVp)	28.25 ± 1.87
Dense	377 (67.32%)	Current (mAs)	98.87 ± 31.86
Sum of MGD (mGy)	6.85 ± 2.90	Compressed breast thickness (mm)	47.94 ± 12.17
Lorad Selenia (*n* = 340)	7.97 ± 2.79	Breast compression force (N)	97.51 ± 28.35
Mammomat Inspiration (*n* = 220)	5.12 ± 2.02	MGD (mGy)	1.81 ± 0.70

Values are given as numbers (%) for categorical variables or means ± standard deviations (ranges) for continuous variables

*MGD* mean glandular dose

**Fig. 1:**
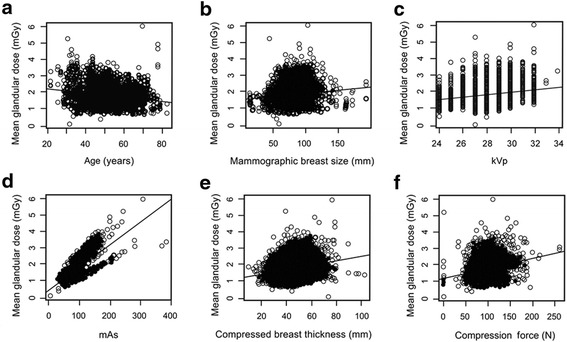
**a** Scatterplot showing a negative correlation between age and MGD. **b**–**f** Scatterplots showing positive correlations between MGD and each of mammographic breast size, kVp, mAs, compressed breast thickness, and compression force

**Fig. 2:**
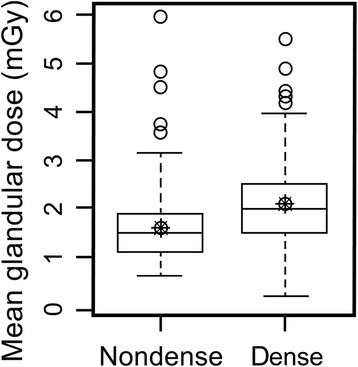
Box-and-whisker plots showing higher MGD in the “dense” group than in the “non-dense” group (1.96 vs. 1.49 mGy)

**Fig. 3:**
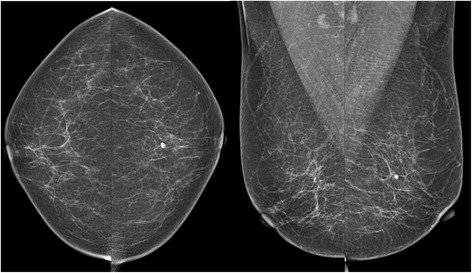
A 62-year-old woman with “non-dense” breast composition and small breasts (mean mammographic breast size, 74.13 mm). The average MGD per view was 1.10 mGy

**Fig. 4:**
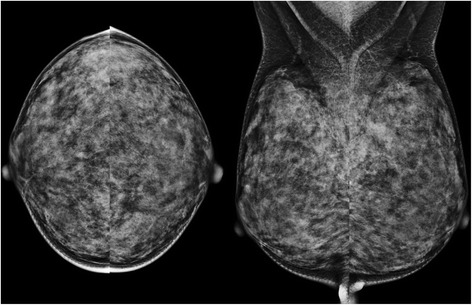
A 41-year-old woman with “dense” breast composition and large breasts (mean mammographic breast size, 83.74 mm). The average MGD per view was 2.38 mGy

In univariate and multivariate linear regression analyses, age was negatively associated with MGD per view. In contrast, the mammographic breast size, mAs, compressed breast thickness, and breast compression force were positively associated with the MGD per view. According to the mammographic breast composition, the “dense” group had a significantly higher MGD than the “non-dense” group (Table 2, Figs. 1, 2, 3, and 4). The average MGD per view in the “non-dense” and “dense” groups was 1.49 and 1.96 mGy, respectively.

**Table 2:** Univariate and multivariate linear regression results of the mean glandular dose per view

	Univariate linear regression			Multivariate linear regression		
	Beta (SE)	*p* value	*R* ^2^	Beta (SE)	*p* value	Adjusted *R* ^2^
Age (years)	−0.013 (0.001)	<0.0001	0.038	−0.002 (0.001)	0.0358	0.820
Mammographic breast size (mm)	0.004 (0.001)	<0.0001	0.016	0.002 (0.0004)	<0.0001	
Mammographic breast composition						
Non-dense	Reference		0.094	Reference		
Dense	0.462 (0.031)	<0.0001		0.201 (0.017)	<0.0001	
Breast-conserving surgery						
No	Reference			Reference		
Yes	0.249 (0.046)	<0.0001	0.014	0.038 (0.020)	0.0605	
Peak kilovoltage (kVp)	0.074 (0.008)	<0.0001	0.039			
Current (mAs)	0.014 (0.0004)	<0.0001	0.384	0.014 (0.0002)	<0.0001	
Compressed breast thickness (mm)	0.013 (0.001)	<0.0001	0.053	0.008 (0.001)	<0.0001	
Breast compression force (N)	0.006 (0.001)	<0.0001	0.058	0.003 (0.0003)	<0.0001	

Beta represents the unstandardized regression coefficient. Due to the high multicollinearity of kVp with several thicknesses, we removed kVp from the multivariate linear regression analysis

*SE* standard error

**Fig. 5:**
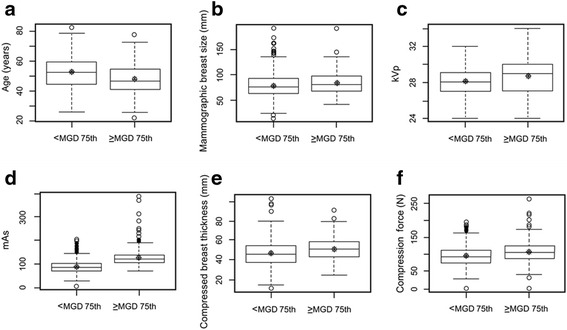
**a**–**f** Box-and-whisker plots showing that doses above the diagnostic reference level are associated with young age, large mammographic breast size, high kVp, high mAs, and increased breast thickness

**Fig. 6:**
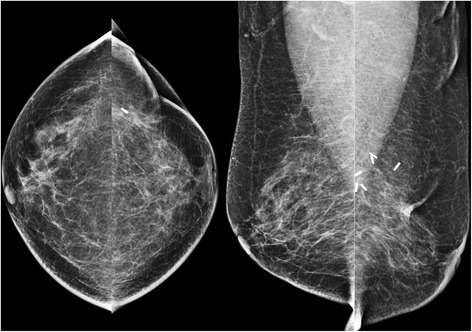
A 69-year-old woman with “non-dense” breast composition, small breasts (mean mammographic breast size, 79.1 mm) and a history of left-sided breast-conserving surgery. The average MGD per view was 1.96 mGy (a higher MGD was observed on the left than on the right side). Left-side average MGD per view, 2.18 mGy; right-side MGD per view, 1.79 mGy

Among the 528 images with doses above the diagnostic reference level, the average MGD was 2.76 mGy. Among the 1592 images with doses below the diagnostic reference level, the average MGD was 1.49 mGy. The mean patient age was younger for images with doses above the diagnostic reference level compared with those below (48.19 vs. 53.17 years, respectively; *p* < 0.0001). The average mammographic breast size was larger in images with doses above the diagnostic reference level compared with those below (84.94 vs. 79.71 mm, respectively; *p* < 0.0001). Images with doses above the diagnostic reference level were more frequently included in the “dense” group of breast composition than were images with doses below the diagnostic reference level (89.77 vs. 59.99%, respectively; *p* < 0.0001). Patients who had doses above the diagnostic reference level more commonly had a history of ipsilateral breast-conserving surgery than did patients who had doses below the diagnostic reference level (18.53 vs. 10.18%, respectively; *p* < 0.0001) (Table 3, Figs. 5 and 6).

**Table 3:** Group characteristics according to the mean glandular dose (75th percentile)

	<MGD 75th percentile	≥MGD 75th percentile	*p* value
View (*n* = 2120)	1592 (75.06%)	528 (24.9%)	
Mean glandular dose (mGy)	1.49 ± 0.43 (0.04–2.28)	2.76 ± 0.47 (2.18–5.97)	
Age (years)	53.17 ± 10.44	48.19 ± 10.83	<0.0001
Mammographic breast size (mm)	79.71 ± 23.45	84.94 ± 19.64	<0.0001
Mammographic breast composition			
Non-dense	637 (40.01%)	54 (10.21%)	<0.0001
Dense	955 (59.99%)	474 (89.77%)	
Breast-conserving surgery			
No	1430 (89.82%)	430 (81.44%)	<0.0001
Yes	162 (10.18%)	98 (18.53%)	
Peak kilovoltage (kVp)	28.09 ± 1.83	28.73 ± 1.91	<0.0001
Current (mAs)	89.60 ± 24.69	126.77 ± 34.65	<0.0001
Compressed breast thickness (mm)	46.86 ± 12.36	51.18 ± 10.98	<0.0001
Breast compression force (N)	94.52 ± 27.06	106.51 ± 30.20	<0.0001

Values are given as numbers (%) for categorical variables or means ± standard deviations (ranges) for continuous variables. *p* values were calculated using independent Student’s *t* tests

*MGD* mean glandular dose

## Discussion

To our knowledge, this is the first study to report the utilization of a radiation dose management system for FFDM in Korean women. Relative to Western women, these women exhibit several differences in breast-related characteristics, including higher rates of dense breasts and small breasts as well as a younger age of breast cancer onset [10–12]. Therefore, the results of this customized study for Korean women provide insight into the factors correlated with the radiation dose, including mammographic breast composition and mammographic breast size. Moreover, the present study describes the present authors’ experience with a commercial radiation dose management system (Radimetrics™) for FFDM. Using this system, we easily collected the MGD values of patients who underwent mammography and analyzed the factors affecting MGD.

In our study, the average MGD per view was 1.81 mGy. This value is similar to values from the American College of Radiology Imaging Network (ACRIN) Digital Mammographic Imaging Screening Trial (DMIST) [7]. Hendrick et al. [13] reviewed the dose data of a large population in ACRIN DMIST and reported an average MGD of 4.7 mGy from two-view screen film mammography and 3.7 mGy from two-view digital mammography. When these MGDs are adjusted by the International Commission on Radiological Protection (ICRP) tissue-weighting factor (0.12 for breast tissue), an average effective dose of 0.56 and 0.44 mSv are obtained [14]. In our study, the average MGD from two-view mammography was approximately 3.62 mGy, which is twice the average MGD from a single view. The average effective dose in our study was 0.43 mSv. This result can be compared to the average effective dose from natural background radiation (3 mSv per year) [15]. In 2007, the ICRP reported that the lifetime attributable risk (LAR) for fatal cancer induction in adults was 4.1% per Sievert [14].

The United States National Academy of Sciences Biologic Effects of Ionizing Radiation (BEIR) VII Group has reported the estimated age-dependent risks of radiation-induced cancer incidence and mortality [3]. Using these BEIR VII estimates, Hendrick [7] reported the age-dependent LAR for two-view mammography with an MGD of 3.7 to 4.7 mGy. For example, the LAR for breast cancer incidence in a 40-year-old woman who has undergone two-view mammography of both breasts is five to seven cases per 100,000. The LAR of a 20-year-old woman is 16–20 cases per 100,000. The LAR of an 80-year-old woman is 0.1–0.2 cases per 100,000. According to this report, even with the same mammographic radiation doses, younger women have a greater risk of developing breast cancer. Furthermore, in our study, young age was significantly associated with high MGD (*p* = 0.0358). Younger women generally have denser breasts and may require higher MGDs. Thus, young age is related to an increased risk of radiation-induced cancer and mortality and is also related to high MGDs. Therefore, better radiation dose management is clearly required for all women, with careful dose management a particularly important consideration for young women undergoing mammography.

The present study also provides insight into the factors that may affect radiation dose. Two major factors that affect radiation dose are the amount of compression and the thickness of the breast [16–19]. In addition to these factors, we assessed mammographic composition and breast size of Korean women. Mammographic breast composition is important for the following reasons. First, dense composition may obscure lesions and lower the mammographic sensitivity. Second, increased mammographic breast composition is a significant risk factor for developing breast cancer [20, 21]. In this study, high MGD was associated with “dense” mammographic breast composition. Ozdemir et al. [22] reported similar results. More radiation penetration is required for dense glandular tissue than for fatty breast tissue. In Korean women, the proportion of “dense” mammographic breast composition based on radiologist estimates and automated volumetric density measurements is 61.9–86.4% (vs. “non-dense”, 13.6–38.2%) [10, 20, 23, 24], whereas in Western women, the corresponding proportion is 36.9–51% (vs. “non-dense”, 49.1–63.2%) [25, 26]. Patients with larger breasts have significantly higher MGDs during mammography. In Korean women, the mean total breast tissue volume according to automated volumetric density measurements is 380.9–466.4 mL [10, 20, 23, 24], whereas the corresponding mean among Western women is 551.95–774 mL [26, 27]. However, in this present study, mammographic breast size was measured from the MLO view. The mean was 82.01 mm, which might be smaller than that of Western women. The linear positive relationship between compressed breast thickness and MGD is well known, and our results are similar to those of prior studies [16–19]. The correlations between mAs and MGD in our study can be explained by the linear increase in dose with mAs, which is related to beam quantity [28].

Furthermore, patients who had undergone breast-conserving surgery required high MGD values. We assume that the postoperative changes, including seroma, hematoma, and surgical clips, cause increased density during ipsilateral mammography.

In a previous study that did not include a radiation dose management system, the identified data were submitted to a medical physicist for data cleaning. The resulting final dataset subjected to analysis predominantly consisted of cases (80% of cases in the dataset) for which technical data were collected [13]. In the present study, although only one institution and two machines were included, there was no data error or data loss. Furthermore, no medical physicist was required for data cleaning.

Our study had several limitations. First, this study included mammographic images from only two manufacturers of one institution, possibly causing patient selection bias. To avoid selection bias, we analyzed the entire data including that for each manufacturer, which could also be a limitation. Second, because of a large number of factors, we did not analyze correlations or all factors. Due to the high multicollinearity of kVp with several thicknesses, we removed kVp as a variable from the multivariate linear regression analyses. There was an inverse association with increased age, which was statistically significant according to univariate analysis but marginally significant after adjusting for other covariates. Our results suggest that breast size and composition are other covariates associated with age; however, further analysis will be needed to confirm this. In addition, we did not analyze other important confounders such as body mass index, and we only analyzed the breast size and thickness. It would be interesting to follow up this study with a larger number of institutions and manufacturers, a correlation analysis, and an examination of confounders.

In centers with good quality control and well-managed devices, such as our center, the radiation dose is not high. Quality control and device management are very important in patient care, and centers and hospitals that use mammography should always endeavor to minimize the radiation dose as much as possible. Furthermore, because mammography is performed only annually or biannually, summation and monitoring with other modalities is needed in future studies.

## Conclusions

Among Korean women, patients with large and dense breasts should be more carefully managed to ensure that a constant radiation dose is maintained. In addition, we should devote particular attention to younger women and patients who have previously undergone breast-conserving surgery. Moreover, a radiation dose management system was useful during FFDM for collecting data and for analyzing factors associated with the radiation dose. We also expect that the radiation dose management system could help control the radiation dose during FFDM.

## Abbreviations

ACRIN: American College of Radiology Imaging Network
BEIR: Biologic Effects of Ionizing Radiation
CC view: Craniocaudal view
DICOM: Digital Imaging and Communications in Medicine
DMIST: Digital Mammographic Imaging Screening Trial
FFDM: Full-field digital mammography
ICRP: International Commission on Radiological Protection
LAR: Lifetime attributable risk
MGD: Mean glandular dose
MLO view: Mediolateral oblique view
PACS: Conventional picture archiving and communication system
